# Associations between load-velocity profiling and race parameters of elite swimmers in the 100 and 200m freestyle events

**DOI:** 10.3389/fspor.2023.1326106

**Published:** 2023-12-14

**Authors:** Yannis Raineteau, Guillaume Nicolas, Benoit Bideau, Nicolas Bideau, Robin Pla

**Affiliations:** ^1^ M2S Laboratory—Laboratoire Mouvement Sport Santé, Université Rennes 2, Rennes, France; ^2^ Optimization service, Fédération Française de Natation, Clichy, France; ^3^ MIMETIC-Team, INRIA Rennes Bretagne Atlantique, Rennes, France

**Keywords:** swimming technique, propulsion, stroke regulation, load-velocity profiles, competitive swimming

## Abstract

**Introduction:**

Improving swimming performance involves assessments of biomechanical variables of the stroke, and it can be achieved using semi-tethered swimming tests. The aim of this study was thus to investigate the associations between load-velocity (L-V) profiles, from a semi-tethered swimming protocol and race variables in the 100 m and 200 m freestyle events.

**Methods:**

Eight swimmers completed a L-V profiling protocol consisting of four sprints (25 m, 25 m, 20 m, 15 m) against increasing loads (0.1, 2.0, 4.0, 6.0 kg respectively) with complete recovery between repetitions (>5 min). The L-V linear regression was used to estimate maximal velocity (V0) and body mass normalized load (rL0). Race variables such as clean swimming speed (V), stroke rate (SR), distance per cycle (SL) and stroke index (SI) were assessed from video analysis of 100 m and 200 m freestyle events taking place 3–4 days after the L-V protocol.

**Results:**

L-V results showed high levels of speed (mean ± SD: 1.87 ± 0.04 m/s) and heavy maximal relative loads (mean ± SD: 38.5 ± 6.51 as % of body mass). Swimmers also achieved high-level performances in the 100 m (mean ± SD time: 51.95 ± 0.75 s) and the 200 m (mean ± SD time: 113.85 ± 2.67 s). For the 100 m events, the maximal relative load showed strong correlation with performance (*r* = 0.63) whereas trivial correlation was observed for the 200 m events (*r *= 0.12). SR on the 100 m and the 200 m also showed very strong association with rL0 (*r* = 0.83) and a strong association with V0 (*r* = 0.68) respectively.

**Conclusion:**

The relationships between L-V variables and race variables depend on the distance of the event. However, L-V variables seem to be less related to SR and SL evolutions for the 100 m than in the 200 m event. Moreover, L-V profiles tend to be more related to the 100 m than 200 m freestyle performance. L-V profile should be interpreted taking into consideration the specific physiological and biomechanical constraints of the main events of the swimmer.

## 1. Introduction

For several years now, with the help of the development of new technologies, the number of studies related to the analysis of competitive swimming performance has increased (1). Some of the variables most commonly used to assess swimming performance include stroke variables such as stroke rate (SR) and distance per cycle (SL) (2–4). However, it is difficult for coaches to have additional monitoring tools during competition. That is why some testing evaluations are needed to assess swimming technique before competition (5).

Among the monitoring tools commonly used in swimming, semi-tethered methods represent a good way to assess biomechanical skills of the swimmers (6–8). Besides, the semi-tethered protocol allows to better understand propulsion (9) and active drag (10). More recently, with the help of technologies (i.e., speedometer and force transducer) resulting in the development of new devices (e.g., SwimOne; Sportmetrics S.L., Spain; 1080 Sprint, 1080 Motion, Lidingö, Sweden), some studies highlighted the possibilities of assessing dynamic variables with an easy and rapid feedback on the field (11–13). Other variables such as the intra-cycle velocity variations (IVV), which is associated to dynamic variables and especially to drag, can also be measured using such devices, providing important additional information (14, 15). Some other studies also investigated the relationships between both propulsive and resistive force, and swimming performance (16, 17). Load-velocity (L-V) profiles can provide information on these aspects of propulsive and resistive forces (9), mainly highlighted by variables such as estimated maximal tethered load (L0) and maximal velocity (V0). These variables correspond to the endpoints of the L-V linear regressions obtained from velocity measurements recorded on sprints against different resistances called “load” and applied with specific devices. Additionally, recent literature demonstrated the strong relationships of L-V profiles with dryland variables (18), and sprint performance (19, 20). Those same authors pointed out the importance of assessing drag and propelling efficiency, to have a complete profile of the swimmer technique. However, the relationships between L-V profile and performance in competition remain unclear.

In other sports such as running, rugby and soccer, force-velocity profiles are one of the major tools to monitor and guide training. By using force-velocity profiles, coaches can design more precise and individualized training programs for each athlete, following a fine-tune methodology (21). This interaction has already been described in other sports such as running and team sports (22–24). Such an individualized approach already demonstrated its efficacy in high level rugby athletes, who improved their capacities after a specific training guided by force-velocity profiles, with resisted or overspeed training (25). Although several studies have described some of these aspects, L-V profiling in swimming is a different concept and it seems important to study it in more detail on very high-level swimmers. The aim of this study was thus to investigate relationships between L-V profiles, evaluated using semi-tethered swimming, with 100 m and 200 m freestyle performances and stroke variables.

## 2. Methods

### 2.1. Participants

Eight male swimmers (18.6 ± 1.9 years, 74.5 ± 5.9 kg of body mass, 182.0 ± 2.9 cm of height) who were part of the same training group voluntarily participated in this study. They swam 1,844 km in the season during which the study was conducted. 62.5% of the annual swimming volume was performed at low intensity [blood lactate (La)_b_ < 2 mmol·L^−1^], 20.5% at moderate intensity [(La)_b_ between 2 and 4 mmol·L^−1^], and 17% at [La]_b_ > 4 mmol·L^−1^ (26). The swimmers had at least 9 years of experience as competitive swimmers and their performance characteristics are presented in Table 1. Based on their personal best performances, seven swimmers can be classified as “level 2” and one as “level 1” (27). The study was conducted in agreement with the Declaration of Helsinki. After a comprehensive oral explanation, all participants signed an informed consent form to participate.

**Table 1:** Information and performance characteristics of the swimmers.

	Age (year)	Height (cm)	Body mass (kg)	Best all-time performance (World Aquatics points)
Swimmer 1	19	183	86.6	737
Swimmer 2	18	178	69.8	771
Swimmer 3	17	185	75.8	766
Swimmer 4	21	180	72.7	930
Swimmer 5	20	186	74.0	807
Swimmer 6	17	182	74.2	788
Swimmer 7	22	180	68.5	851
Swimmer 8	20	185	78.6	861

### 2.2. Experimental overview

The testing session took place before an international meeting. The L-V profiling was performed in a 25-m indoor swimming pool (air temperature: 25.1°C, water temperature: 28.5°C). Swimmers were equipped with their usual training suit. The testing session was conducted between 7:00 and 9:00 am on the same day for every swimmer. They performed an individualized warm-up of 20 min including 600 m at low intensity, 200 m of specific drills and three repetitions of short distances (20 m) at maximal speed. The competition took place 3 days (for the 100 m) and 4 days (for the 200 m) after the testing session. One swimmer did not take part in the 100 m freestyle event, and another swimmer did not take part in the 200 m freestyle event.

### 2.3. Testing session

The swimmers performed a resisted sprint protocol, using the 1080 motion (Lidingö, Sweden) allowing to complete a L-V profiling. The testing procedure was already described in a previous study (19). The protocol consisted of four all-out sprints: 25 m, 25 m, 20 m, and 15 m against increasing loads (0.1, 2.0, 4.0 and 6.0 kg respectively) with complete recovery (>5 min). Instantaneous speed was collected directly via the device for each imposed load. Regarding post-session data processing, average swimming velocities were computed using three cycles in the middle of the sprints. Maximum values of velocity (V0), body mass normalized load (rL0) and the Slope were computed using the L-V linear regression. Arm stroke Froude efficiency (ηF) was calculated using the following equation (28), assuming that the arm stroke contributed to 90% of the speed in a 25 m all-out sprint, as previously suggested (29, 30):

$$\eta F=\frac{0.9\;\text{·}\upsilon }{\upsilon_{\mathrm{hand}}}$$

where *v* is the speed of the swimmer and *v*_hand_ is the effective hand speed calculated according to a model proposed in a previous study (31). Finally, Intracycle Velocity Variation (IVV) was computed using this equation (32):

$$\mathrm{IVV}=\frac{\mathrm{Standard}\;\mathrm{deviation}\;\mathrm{of}\;\mathrm{velocity}}{\mathrm{mean}\;\mathrm{velocity}}\;\times 100$$

### 2.4. Video recording

During the competition, all the events were recorded with one fixed 1080p camera (Sony FDR AX700) in panoramic mode, which was positioned at the 25 m mark on the top of the stands and perpendicularly to the long axis of the pool. The sampling rate was 50 Hz. As described in a previous study (33), the video was then analyzed frame by frame using a dedicated software to calculate speed and stroke variables for the swimmer.

### 2.5. Data processing and analysis

Race analysis software (Actriss, Brest, France) was used for calibration and image processing as already described (4). Using manual digitalization with the lane markers located every five meters (34), the video analyst annotated the time when the head of the swimmer crossed each lane marker, and at the beginning of each cycle. To measure all stroke variables, the time, and points of the first and last arm entry for each lap was calculated, giving the beginning and the end of each “free-swimming” period, and allowing to compute clean swimming speed (V; swimming speed excluding underwater parts) as depicted in (33). This clean swimming speed (m/s) was determined by dividing the total distance covered during the free-swimming period per the time spent during that free-swimming period. SC is the total number of arm entries on the water surface. SL is calculated dividing the free-swimming distance by SC. SR is calculated dividing the free-swimming time by SC. SI is the product of the swimming speed (lap distance divided by lap time) and SL. We collected those stroke variables for each lap (one value per lap for each stroke parameter). For statistical analysis, we calculated an overall mean for the race for each stroke variable (using the mean of the two laps for the 100 m and the four laps for the 200 m). All analysis were conducted by the same experienced analyst who worked for the French swimming federation.

### 2.6. Statistical analyses

For all variables, descriptive statistics (mean, standard deviation, minimum, maximum and variation coefficient) were performed. Shapiro–Wilk test was used to verify the normality of the data. R coefficients of Pearson correlations were computed between L-V and race variables. We considered correlation threshold values of 0.1, 0.3, 0.5, 0.7, and 0.9 corresponding to small, moderate, strong, very strong, and nearly perfect correlations, respectively (35). Statistical significance of the data was assessed using the Student's *t*-test. Null hypothesis was rejected at *p* < 0.05. Statistical analyses were undertaken using the Rstudio software package (PBC, Boston, Massachusetts, USA).

## 3. Results

Swimmers performed an average time of 51.95 s (minimum: 50.89 s; maximum: 52.47 s) in the 100 m freestyle and 113.85 s (minimum: 109.55 s; maximum: 117.73 s) in the 200 m freestyle, corresponding to 90.3% and 89.6% to the current world records respectively.

**Figure 1:**
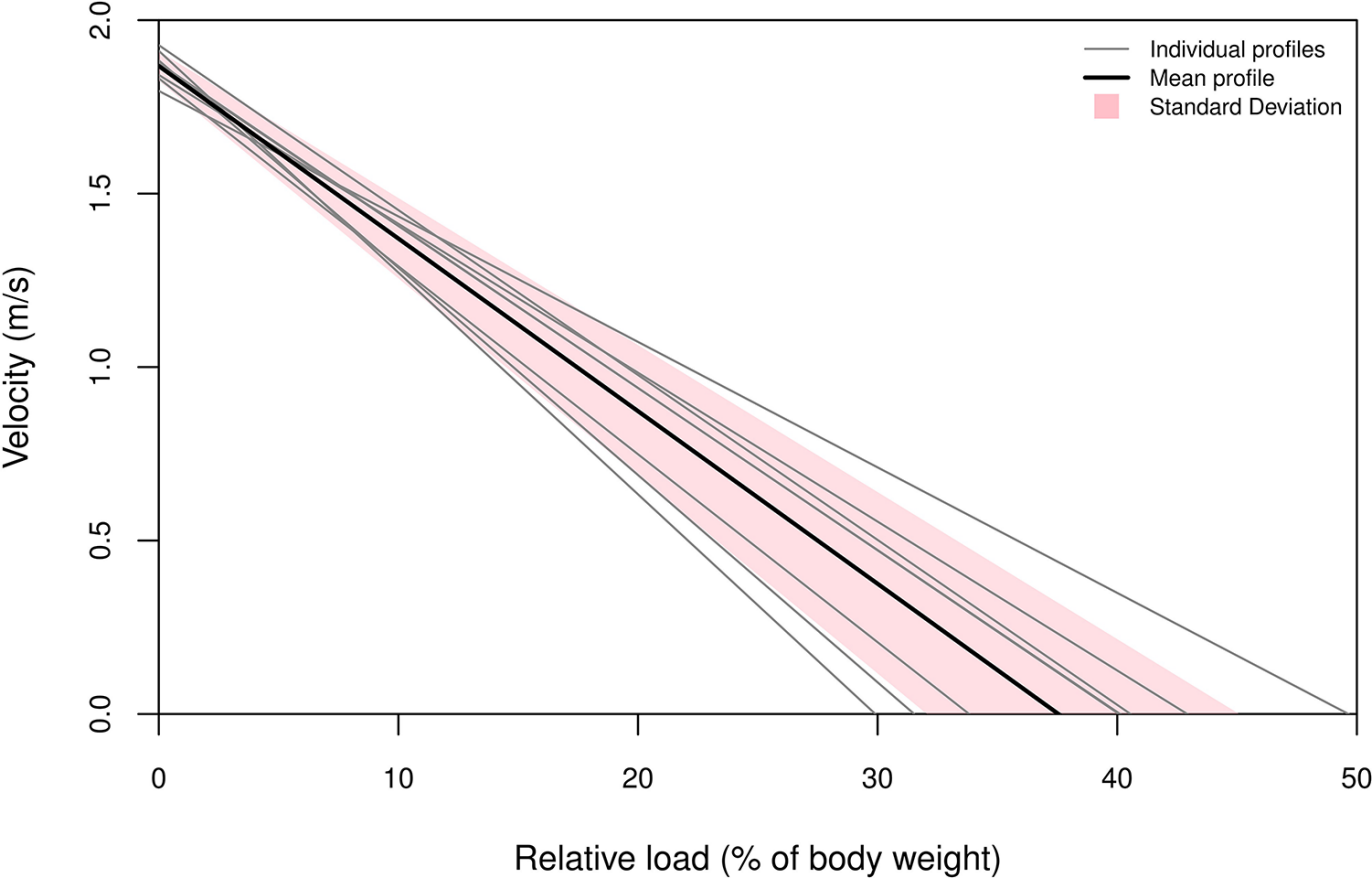
Results of individual load−velocity linear regressions, presenting also the inter-individual mean and standard deviation of the load-velocity group results.

Despite homogeneous results in competition, swimmers showed a wide range of L-V profiles (Figure 1). For a small range of V0 (between 1.80 m/s and 1.93 m/s), we observed a wide range of rL0 (from 0.30 to 0.50 kg/kg of body weight). V0 and IVV were the variables with the lowest coefficient of variation, reflecting more homogeneous results (Table 2). All stroke variables are presented for the 100 m and 200 m freestyle (Table 2). The values are the intra-individual averages of these variables over the whole race. We observed more variations of SL than SR during both the 100 m and 200 m freestyle events.

**Table 2:** Load-velocity results and race parameters.

	Mean ± SD	Min	Max	Inter-individual coefficient of variation
Load-velocity results				
Estimated maximal velocity (m/s)	1.87 ± 0.04	1.80	1.93	2.30%
Estimated maximal relative load (% BW)	38.5 ± 6.51	29.9	49.7	16.90%
Arm stroke efficiency (% Wt)	51.0 ± 3.8	46	56	7.48%
Intracycle velocity variation (% Vmean)	10.7 ± 2.77	6.1	14.8	25.98%
100 m freestyle race parameters				Mean of intra -individual coefficient of variation (by 50 m laps)
Clean swimming speed (m/s)	1.79 ± 0.03	1.76	1.83	1.66%
Stroke rate (cycle/min)	49.6 ± 0.9	48.3	50.6	1.90%
Stroke length (m/cycle)	2.24 ± 0.05	2.17	2.32	3.31%
Stroke index (m^2^/s)	4.02 ± 0.12	3.82	4.16	4.79%
200 m freestyle race parameters				
Clean swimming speed (m/s)	1.73 ± 0.04	1.67	1.79	1.97%
Stroke rate (cycle/min)	42.9 ± 2.2	39.9	45.6	4.18%
Stroke length (m/cycle)	2.39 ± 0.13	2.24	2.53	5.40%
Stroke index (m^2^/s)	4.13 ± 0.27	3.80	4.52	6.22%

SD, standard deviation; Min, minimal value of the eight swimmers; Max, maximal value of the eight swimmers.

**Figure 2:**
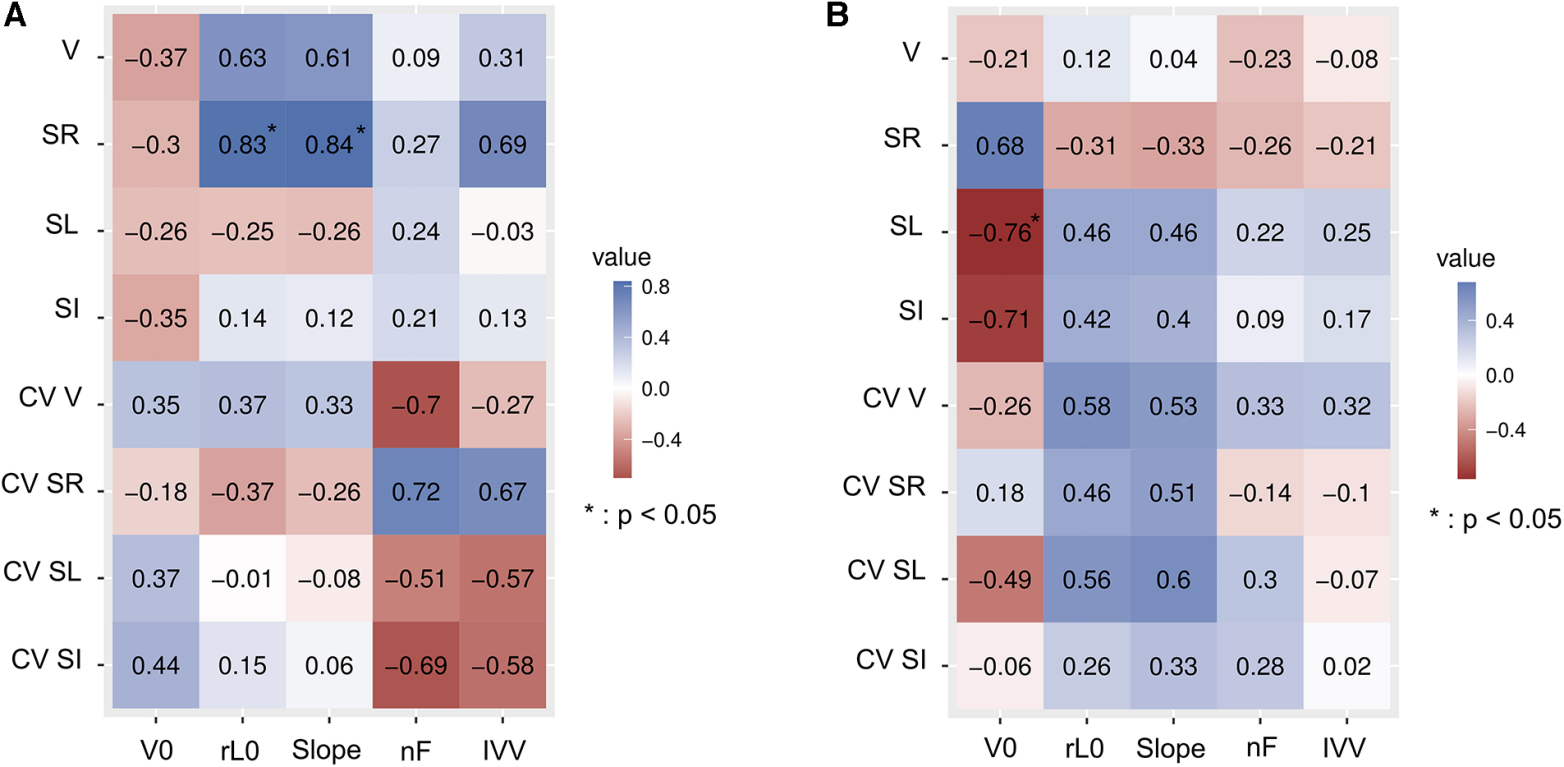
(**A**) *R* coefficients of Pearson correlations between 100 m race parameters and parameters obtained from the testing session. V, clean swimming speed; SR, stroke rate; SL, stroke length; SI, stroke index; CV, intra-individual coefficient of variation (for V, SR, SL and SI variables); V0, estimated maximal velocity; rL0, estimated maximal relative load; Slope, slope of the L-V linear regression; nF, arm stroke efficiency; IVV, intracycle velocity variation. (**B**) *R* coefficients of Pearson correlations between 200 m race parameters and parameters obtained from the testing session. V, clean swimming speed; SR, stroke rate; SL, stroke length; SI, stroke index; CV, intra-individual coefficient of variation (for V, SR, SL and SI variables); V0, estimated maximal velocity; rL0, estimated maximal relative load; Slope, slope of the L-V linear regression; nF, arm stroke efficiency; IVV, intracycle velocity variation.

**Figure 3:**
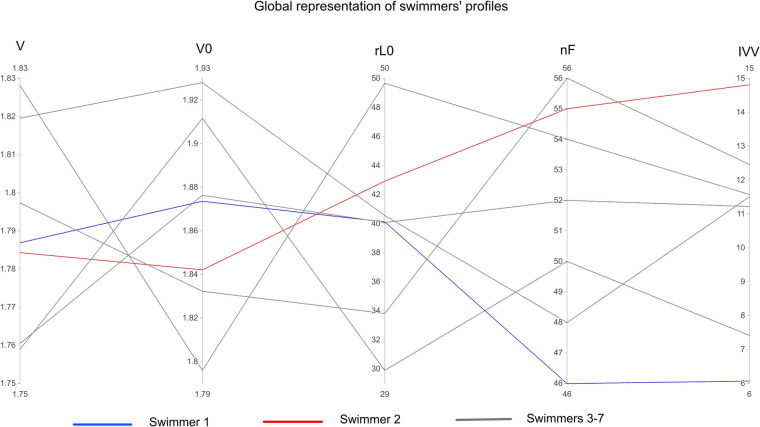
Global representation of swimmers’ profiles, considering the speed variable during the 100 m freestyle event and other variables from the testing session. Each line represents one swimmer. V, clean swimming speed during the 100 m (m/s); V0, estimation of maximal speed (m/s); rL0, estimation of maximal relative load (as % of body mass); ηF, arm stroke efficiency (as % of total arm stroke power production); IVV, intracycle velocity variation (as % of mean velocity).

Matrix of correlation (Figures 2A,B) described the relationships between L-V profiles and stroke variables for 100 m and 200 m freestyle respectively. V0 had a very strong correlation with SL on the 200 m but only small or moderate correlations were found between V0 and race variables on the 100 m. On another hand, rL0 had a very strong correlation with SR and a strong correlation with the clean swimming speed on the 100 m but only small or moderate correlations were found between rL0 and race variables on the 200 m. Figure 3 represented the profiles of two different swimmers with similar 100 m performances but with different hydrodynamic profiles.

## 4. Discussion

### 4.1. Originality of the study

This study is the first to describe associations between in-water L-V profiles and race variables of elite swimmers. The main findings suggest that V0 was not a good determinant of 100 m nor 200 m performance but was strongly related to race kinematics (SL, SR, and SI) whereas rL0 was strongly correlated with the performance on the 100 m. This study is also the first to present L-V profiles of swimmers of such level. Indeed, our results showed higher values of V0 and rL0, compared to previous work on elite swimmers (20). Moreover, these results highlighted a more homogeneous group with a lower CV for V0 and rL0 variables. We also observed different findings regarding the correlations between L-V results and the swimming speed compared to the above-mentioned study, both rL0 and V0 showing lower association with performance in the present study. The expression of performance (i.e., clean swimming speed vs. total mean speed) and the distance evaluated (i.e., 100 & 200 m vs. 50 m) differed between the two studies [i.e., the present one and (20) respectively] which could explain the different results.

### 4.2. Relationships between load-velocity results and race variables

Correlations between L-V results and race variables also differed between the two distances evaluated in the present study. The greater the distance, the more stroke variables are associated with V0 (Figure 2). This can be explained by the fact that both distances require specific physiological and biomechanical abilities (1). However, V0 was not correlated with 100 m and 200 m performance. For 100 m, we observed a strong correlation between V and rL0 and a very strong correlation between SR and rL0. This in line with a previous study (36) which showed similar results, highlighting a strong correlation between mean force values during 30 s tethered swimming and 100 m performance (*r* = 0.72). Moreover, the association between rL0 and SR in the 100 m could be explained by the ability of short-distance swimmers to produce more force and power, and probably to maintain a higher SR (37). Other studies also showed that an improvement of SR and velocity could occur after a strength training program (38). Moreover, we already know that elite swimmers demonstrate a higher SR than the others, and it is associated with high propulsive phase and by the stability of these values during the race (39).

### 4.3. Relationships between race variables changes and load-velocity results

Correlations between the different biomechanical variables and variations of speed and stroke variables over races support this observation. We observed that ηF in the testing session presented a negative correlation with variations of speed in the 100 m, while it was positively correlated with variations of SR in the same event. We can then deduce that the most efficient swimmers seem to maintain their speed all along the 100 m distance using the ability to adjust their SR. For comparison, a previous study showed that the fastest swimmers in the 100 m freestyle were characterized by the capacity to maintain their stroke variables throughout the race (40). The results of this study differ slightly from ours, but it should be noted that the considered variables are not the same (i.e., efficiency vs. velocity). Indeed, ideally the ability to swim fast arises from the capacity: (i) to produce a high mechanical power output enabling the generation of high propulsive force; (ii) to reduce drag; (iii) to perform at a high efficiency (41). Moreover, some studies showed most efficient swimmers are also more economical (42) which may be beneficial to maintain speed throughout 200 m but also 100 m.

The relationships between evolutions of speed and stroke variables with biomechanical variables such as the tethered force were also investigated by some authors (43) who have shown a strong correlation (*r* = 0.62) between the peak force (Fpeak) and the relative changes in velocity during the 100 m freestyle. We found a lower association between rL0 and velocity variations over the 100 m. We speculate that the difference in results is given to the different dynamic parameters measured in both our study (rL0) and the above-mentioned study (Fpeak), suggesting that rL0 would reflect different qualities compared to Fpeak.

### 4.4. Profile overview

This hypothesis may be supported by the case study of two swimmers who achieved similar 100 m freestyle performances (i.e., 49.04 and 49.37 s, cf. Figure 3). They also had similar L-V results, yet each had very different hydrodynamic profiles representing for some variables the opposite extreme values measured on this group. Indeed, we can see that swimmer 1 is able to compensate for its lower propulsive efficiency with lower IVV and therefore lower active drag as assumed (15). This highlights the importance of combining measurements, to assess kinematic and dynamic variables in swimming.

### 4.5. Limitations

These results must be considered acknowledging the potential shortcomings of this study. First, the number of participants was limited, and the population included only male swimmers, for which we have only analyzed their races in front crawl. Moreover, the evaluation and the competition occur at a time of their season when they were not in their best shape, and the group's coach had not tapered them to perform at this competition. All these reasons may have counterbalanced some conclusions of this study.

## 5. Practical applications

The findings of this study propose implications for coaches who are sensitive to the assessment of swimmers' technique during training periods. L-V profiling, and the biomechanical variables derived from it, can help to evaluate technique, track changes for stroke variables and identify areas for improvement in both propulsion and technical aspects of front crawl. Then, profiling the swimmer through this semi-tethered sprint protocol provides a comprehensive swimmer's identity card. Furthermore, maximal relative load representing high degree of resistive swimming and being associated with performance on several distances could justify the use of in-water resistance workouts.

## 6. Conclusion

This study shows associations between L-V profiles and race spatio-temporal variables on a small group of swimmers. Those insights revealed the importance to consider a wide range of biomechanical variables when assessing a swimmer's profile. This study also highlighted the diversity of each race distance in many aspects, which seems to have an impact on the relationship between L-V profiling results and race variables.

## Data Availability

The raw data supporting the conclusions of this article will be made available by the authors, without undue reservation.
